# Attentional bias toward emotional faces in cognitive disengagement syndrome

**DOI:** 10.3389/fpsyg.2026.1855003

**Published:** 2026-09-07

**Authors:** Eun-Bin Shin, Yu-Jin Choi, Gi-Eun Lee, Jang-Han Lee

**Affiliations:** Department of Psychology, Chung-Ang University, Seoul, Republic of Korea

**Keywords:** ADHD, attentional bias toward emotional faces, attentional processing of emotional information, cognitive disengagement syndrome, eye-tracking

## Abstract

**Introduction:**

Given the conceptual overlap yet increasing distinction between cognitive disengagement syndrome (CDS) and Attention Deficit/Hyperactivity Disorder (ADHD), this study examined attentional bias toward emotional faces in adults with CDS and compared their patterns with those of individuals with ADHD to clarify distinct attentional processing profiles.

**Methods:**

Following an initial screening of 1,098 adults using the Barkley Adult ADHD Rating Scale and the Adult Concentration Inventory, 96 participants were recruited and categorized into four groups: CDS only (*n* = 35), ADHD only (*n* = 21), comorbid CDS and ADHD (*n* = 17), and a control group with neither condition (*n* = 23). Participants completed a free-viewing eye-tracking task in which pairs of emotional and neutral faces (angry–neutral, sad–neutral, happy–neutral) were presented. Attentional bias was analyzed by distinguishing early orienting (proportion of first fixation) and later attentional maintenance (total dwell time).

**Results:**

Results indicated that individuals with CDS (CDS only and CDS+ADHD groups) showed a trend toward a lower proportion of first fixations toward angry faces as compared to those without CDS. In addition, individuals with CDS exhibited longer dwell times for all emotional faces. In contrast, individuals with ADHD symptoms (ADHD only and CDS+ADHD groups) demonstrated greater dwell time specifically for angry faces as compared to those without ADHD symptoms.

**Discussion:**

These findings suggest that CDS may be associated with difficulty detecting angry faces at the early stage of attentional processing and with generalized difficulty disengaging from emotional faces at the later stage. By contrast, ADHD symptoms may be selectively associated with anger-specific attentional bias at the later stage. Overall, the present study suggests that CDS and ADHD may be characterized by distinct patterns of attentional processing in response to emotional faces.

## 1 Introduction

Cognitive disengagement syndrome (CDS), formerly known as sluggish cognitive tempo (SCT), is characterized by a range of developmentally inappropriate cognitive and motor symptoms that are linked to functional impairments and often co-occur with other psychological disorders (Becker, 2025). The cognitive features manifest as difficulties in maintaining engagement with the external environment. More specifically, this can manifest as slowed thinking and processing, frequent daydreaming, mental fogginess, losing track of one’s thoughts, blank staring, confusion, and a general sense of apathy or lack of motivation (Barkley, 2014; Fredrick and Becker, 2023). Although CDS was initially identified in studies of ADHD, it has increasingly been conceptualized as a construct distinct from ADHD symptoms, with strong independent support in the research literature (Barkley, 2013, 2016; Becker et al., 2016, 2023). Beyond these attentional and cognitive characteristics, accumulating evidence suggests that CDS is also closely associated with emotional functioning, particularly internalizing difficulties.

According to prior research, individuals with CDS symptoms have been reported to experience more severe difficulties in emotion regulation than individuals with ADHD symptoms (Barkley, 2012; Yılmaz and Bahadır, 2026). CDS is strongly associated with internalizing symptoms such as anxiety and depression, and these associations remain significant even after controlling for ADHD inattention symptoms (Becker et al., 2020; Lee et al., 2014). Emotion regulation difficulties related to CDS have been linked to social impairments, including social withdrawal, isolation, and reduced initiative (Fredrick and Becker, 2022; Miller et al., 2024; Willcutt et al., 2014). In contrast, ADHD symptoms—particularly those related to hyperactivity and impulsivity—have been found to be more closely associated with externalizing problems such as anger and aggression (Barkley, 2014; Lee et al., 2014). These findings suggest that while both disorders involve emotion regulation difficulties, they may differ qualitatively in their manifestation and predominant emotional challenges (Cano-Crespo et al., 2024).

According to the process model of emotion regulation, regulatory processes operate at each stage of emotion generation (Gross and Thompson, 2014), and emotions can be modulated by rapidly shifting attention toward alternative external stimuli (Sheppes and Gross, 2012; Van Dillen and Koole, 2007). In ADHD, difficulties in attentional allocation and inhibitory control in response to emotional stimuli have been linked to emotion dysregulation (Rubia et al., 2009; Shaw et al., 2016). These processes rely on executive functions that enable the suppression of task-irrelevant information and flexible attentional shifting; inefficiencies in these later-stage control mechanisms have been associated with disengagement difficulties (Park and Lee, 2021; Weigard et al., 2018).

Given that CDS has been conceptualized as an attention-related condition (Barkley, 2014), its characteristic attentional profile may likewise influence emotion regulation. Prior research suggests that individuals with CDS show reduced efficiency in the orienting network (Becker and Willcutt, 2018; Kim and Kim, 2021). The perceptual decoupling model proposes that excessive allocation of attentional resources to internally generated thought may limit the resources available for processing external stimuli (Schooler et al., 2011), potentially resulting in inefficient initial orienting to emotional cues. In addition, emerging findings indicate that individuals with CDS may also experience difficulties disengaging attention from threat-related stimuli (Kim and Lee, 2025), suggesting that both early and later stages of selective attention could be implicated.

Taken together, CDS may involve difficulties at both the initial orienting stage and the later reallocation (disengagement) stage of selective attention, potentially resulting in inefficiencies across the overall processing of emotional stimuli. Given the limited capacity of attentional resources, the selective allocation of attention to emotional information plays a central role in generating, modifying, and attenuating emotional responses (Koster et al., 2009). Accordingly, inefficiencies in attention–emotion interactions may negatively influence the regulation of emotional responses. However, relatively few studies have directly examined the link between attentional characteristics and emotional processing in individuals with CDS. Therefore, distinguishing the stages at which attentional biases emerge in CDS represents a theoretically meaningful endeavor.

Attentional bias can arise from two distinct mechanisms: (a) initial orienting toward a stimulus or (b) difficulty disengaging attention from a selected stimulus and reallocating it elsewhere (Posner et al., 1987). Initial orienting is considered an early, stimulus-driven selection mechanism that operates independently of intention (Awh et al., 2012) and typically occurs within approximately 150 ms. In contrast, difficulty disengaging attention from a selected stimulus reflects a later-stage mechanism emerging around 200–250 ms after stimulus onset (Weierich et al., 2008).

In the present study, emotional stimuli consisted of facial expressions. Because facial expressions play a crucial role in identifying emotional states and facilitating social interaction, attentional bias toward emotional facial expressions has been extensively studied. This is because emotional faces convey salient information related to friendliness, emotional cues, and social value (Hare et al., 2005). According to studies on the attentional processing of emotional faces in psychiatric patient populations, attentional bias toward emotional faces has been shown to predict the onset, persistence, and exacerbation of negative emotional symptoms (Vazquez et al., 2016). Individuals exhibiting internalizing symptoms, such as depression or anxiety, tend to show an attentional bias toward faces associated with their symptoms (e.g., sad faces) (Cisler and Koster, 2010). Individuals with externalizing traits, such as ADHD, are known to have difficulty inhibiting attention toward angry faces (Manoli et al., 2021).

Separating attentional bias toward emotional faces into these two mechanisms is useful for clarifying how emotional face processing operates in CDS. This may help distinguish its attentional profile not only from that of ADHD but also from profiles associated with depression or anxiety. To examine attentional bias toward emotional faces at a stage-specific level, it is necessary to employ appropriate analytical measurement methods. Eye-tracking technology provides a detailed analysis of attentional components—from initial orienting to attentional disengagement—across extended time intervals during naturalistic information processing. Because gaze direction is closely linked to attentional focus, eye-tracking allows for the separate assessment of early orienting and later disengagement mechanisms of attentional bias toward emotional faces. For this reason, eye-tracking is particularly useful, as behavioral measures alone may not sufficiently capture the temporal dynamics of attention (Wright and Ward, 2008).

Therefore, the primary aim of the present study was to examine attentional bias toward emotional faces in individuals with CDS and to compare their attentional profile with that of individuals with ADHD. To this end, participants were categorized based on the presence or absence of CDS symptoms into a CDS group (CDS only and ADHD+CDS) and a non-CDS group (ADHD only and healthy controls). It was hypothesized that individuals with CDS symptoms would exhibit greater deficits in initial orienting toward emotional faces compared to individuals without CDS symptoms. Furthermore, with respect to difficulties in attentional disengagement, individuals with CDS were expected to demonstrate impaired shifting of attention away from emotional stimuli, resulting in a late-stage attentional bias across emotional face types. In contrast, individuals with ADHD were expected to show greater difficulty disengaging attention specifically from angry faces, consistent with increased challenges in anger and aggression regulation. Accordingly, it was hypothesized that the ADHD group would exhibit a late-stage attentional bias selectively toward angry faces, rather than across all emotional expressions.

## 2 Materials and methods

### 2.1 Participants

Participants, young adults with attentional problems, were recruited through online communities, the Internet bulletin board of several universities and the advertisements in psychiatric clinics in Seoul, Korea. A total of 1,098 adults were initially screened using the Barkley Adult ADHD Rating Scale IV (BAARS-IV; Barkley, 2011) and Adult Concentration Inventory (ACI; Becker et al., 2015). Based on the previous inclusion criteria (Barkley, 2011, 2012; Becker and Barkley, 2018), a symptom threshold of five or more SCT symptoms and four or more ADHD symptoms was used. These thresholds correspond approximately to the 95th percentile in community samples and were coupled with evidence of self-reported impairment in one or more major life activities. The following four groups were created: (a) high levels of both CDS and ADHD; (b) high levels of CDS but not ADHD; (c) high levels of ADHD but not CDS; and (d) healthy controls. The ADHD groups (ADHD-only and CDS+ADHD) included participants meeting the BAARS-IV screening criteria for inattentive, hyperactive-impulsive, and combined ADHD presentations. A total of 114 participants were screened using the structured clinical interview for DSM-5 (SCID-5; First et al., 2016). Additionally, participants were asked to refrain from taking any medication on the day of participation, as this would allow their dysfunction to be more accurately measured. Of the 114 participants, 18 participants were excluded – five participants with other psychiatric disorders, 10 participants whose gaze percent was too low to allow for eye-tracking data to be analyzed and three participants whose eye-tracking measurements deviated less or more than 2SD from the mean. Thus, a total of 96 participants were accepted into the analysis. The groups were divided into 17 individuals with CDS+ADHD, 35 individuals with CDS only, 21 individuals with ADHD only, and 23 healthy controls. All participants had normal or corrected-to-normal vision.

### 2.2 Materials

#### 2.2.1 The Barkley Adult ADHD Rating Scale IV (BAARS-IV)

The BAARS-IV, which was originally developed to assess the levels of ADHD and CDS (Barkley, 2011) and was validated (Becker et al., 2014), contains 18 items that are consistent with DSM-5 criteria for ADHD, and 9 items that target the symptoms of CDS. Using a four-point Likert scale (1 = not at all; 2 = sometimes; 3 = often; 4 = very often), the participants responded to each item with reference as to how often each statement best described their behavior in the past 6 months. In the present study, Cronbach’s α values were 0.88, 0.88, and 0.89 for the subscales of ADHD inattention, ADHD hyperactive-impulse, and CDS, respectively.

#### 2.2.2 The Adult Concentration Inventory (ACI)

The ACI, developed for a new adult self-report measure of CDS (Becker et al., 2015), includes 16 items identified in a recent meta-analysis as being optimal for the assessment of CDS symptoms (Barkley, 2016; McBurnett et al., 2014). These items were rated on a four-point scale (0 = not at all; 1 = sometimes; 2 = often; 3 = very often) with reference to the past 6 months. In this study, Cronbach’s α was 0.90.

#### 2.2.3 The beck depression inventory-second edition (BDI-II)

The BDI-II, developed to assess the levels of depression (Beck et al., 1996), includes 21 items associated with physical and cognitive symptoms of depression. These items were rated on a four-point scale (0 = not at all; 1 = mildly; 2 = moderately; 3 = severely) with reference to 1 week. In this study, Cronbach’s α was 0.93.

#### 2.2.4 The beck anxiety inventory (BAI)

The BAI, developed to assess the levels of anxiety (Beck et al., 1988), includes 21 items related to physical and cognitive symptoms of anxiety. These items were rated on a four-point scale (0 = not at all; 1 = mildly; 2 = moderately; 3 = severely) with the reference to 1 week. In the present study, Cronbach’s α was 0.93.

#### 2.2.5 The Wechsler adult intelligence scale-fourth edition (WAIS-IV)

The WAIS-IV was developed as a measure of general intellectual functioning (Wechsler, 2008). In the present study, the Arithmetic and Information subtests of the WAIS-IV were used, as these subtests were reported to have the strongest correlation with the full scale intelligence quotient (IQ) in the Korean WAIS-IV as a screening measure of intelligence. Cronbach’ α values were .83 for the Arithmetic and .96 for the Information subscale. The estimated full-scale IQ was calculated using regression equations [54.762 + (2.330 × AR) + (2.151 × IN)] suggested elsewhere (Choe et al., 2014).

#### 2.2.6 The positive affect and negative affect scale (PANAS)

The PANAS, developed to assess individuals’ mood (Watson et al., 1988), includes 20 items measuring the positive affect and negative affect factors. These items were rated on a five-point scale (1 = not at all to 5 = very highly). In this study, Cronbach’s α values were 0.87, 0.90 for positive and negative affect separately.

### 2.3 The attentional bias task

**FIGURE 1:**
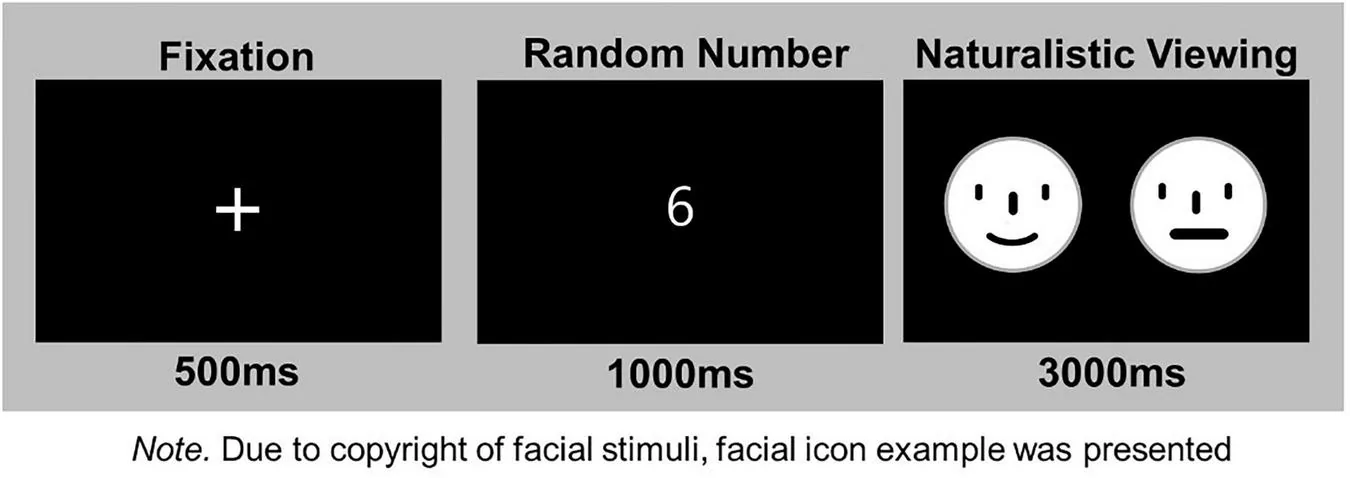
The example of presenting a happy emotional face in the attentional bias task.

The attentional bias task consists of 108 trials with a free-viewing paradigm (Sanchez et al., 2013). Each trial starts with a black screen for 500 ms followed by a white fixation cross displayed at the center of the screen for 500 ms. Thereafter, a random white number (1–9) is displayed. Participants are asked to say the number as quickly as possible to ensure that participants maintain their attention on the fixation cross (Calvo and Avero, 2005). Afterwards, a pair of emotional faces, namely images of an emotional (either happy, sad, or angry) and a neutral facial expression made by the same actor is displayed for 3,000 ms. Participants are instructed to freely look at the faces, without any constraints. Free viewing of image pairs was implemented to encourage naturalistic information processing (Isaacowitz, 2005). Each emotional face will be presented equally often and its position on the screen (right or left side) will be randomly counterbalanced (Figure 1). In total, 36 happy-neutral, 36 sad-neutral, and 36 angry-neutral trials will be ordered in the random order. The facial stimuli in this study were selected from ChaeLee Korean facial expressions of emotions (Lee et al., 2013). The task lasts about 10 min.

#### 2.3.1 Assessment of attentional bias

Attentional bias is assessed by bias score in the proportion and absolute indices by the eye-movement time in seconds (Sanchez et al., 2013). A bias score greater than 0.5 indicates a biased preference to look at the emotional faces rather than the neutral faces. Based on the two mechanisms of attentional bias (Field et al., 2009; Posner et al., 1987), initial orienting and difficulty in disengagement are assessed. Initial orienting is assessed by the initial direction bias score (Duque and Vázquez, 2015). More precisely, the initial direction bias score refers to the proportion of an initial fixation to the emotional face after the picture onset. This is calculated according to the number of trials in which the first fixation was toward the emotional face, which is then divided by the total number of trials with the first fixation toward neutral-emotional face pairs. The other mechanism of attentional bias, difficulty in disengagement, is assessed by bias score in total dwell time and the total dwell time. The bias score in total dwell time refers to the proportion of the mean dwell time, calculated by dwell time to each emotional face divided by total mean dwell time. Meanwhile, the total dwell time toward emotional faces refers to the total mean dwell time that participants fixate on the emotional face during the 3,000 ms period.

### 2.4 Apparatus

The task was presented on a desktop PC using an eye-tracking device (Tobii TX 300, Tobii Technology AB, Danderyd, Sweden). Eye movements were recorded with a sampling rate of 300 Hz. Participants’ eyes were kept 70 cm from the screen with the participants being permitted to naturally move their heads and eyes without any attached sensors during the experiment. Two areas of interest were identified for each trial corresponding with the total area for the emotional and neutral face with Tobii.

### 2.5 Procedure

After they received brief instructions regarding the overall procedure and their rights as research participants, participants signed an informed consent form as approved by the institutional review board of Chung-Ang University (No.1041078-201912- HRSB-363-01). Afterwards, the participants were asked to complete self-report questionnaires. Following this, the procedures of the attentional bias task were implemented. Upon completion of the task, the participants took part in a clinical interview and a standardized intelligence test. Lastly, participants received a debriefing on the purpose of the study and were presented with their small monetary rewards of 10,000 won (ca. 10 USD). It took each participant about 60 min to complete the whole experiment.

### 2.6 Data analysis

To examine the group differences in the characteristics of all groups, a chi-square test and a one-way analysis of variance (ANOVA) were performed. In order to investigate attentional processing of emotional faces, a three-way 2 (CDS: CDS, non-CDS) × 2 (ADHD: ADHD, non-ADHD) × 3 (Emotion: happy, sad, angry) mixed-model ANOVA was conducted. CDS and ADHD were included as between-subject factors, and emotional categories were included as a within-subject factor. When significant main or interaction effects were observed, Bonferroni-corrected *post hoc* pairwise comparisons were conducted. For *post hoc* pairwise comparisons, mean differences (MD), standard errors (SE), and Bonferroni-adjusted *p*-values are reported. In addition, to assess whether there were any significant effects in ANOVAs, an analysis of covariance (ANCOVA) was also performed with depression and anxiety being the covariates. Further, a series of bivariate correlation analyses were performed to investigate associations between CDS symptoms and attentional indices. All statistical data were analyzed using SPSS 20.0 for Windows.

## 3 Results

### 3.1 Demographic and clinical characteristics

The demographic and clinical characteristics of the participants were analyzed (Table 1). This revealed no significant differences in the mean age [*F*(3, 95) = 0.77, *n.s.*], the proportion of sex [χ^2^(3) = 1.85, *n.s*.], or the estimated IQ [*F*(3, 95) = 2.30, *n.s.*] between the groups.

**TABLE 1:** Demographic and clinical characteristic of each group.

Measure	CDS		Non-CDS		Test statistics (F/χ^2^ )
	ADHD	Non-ADHD	ADHD	Non-ADHD	
	CDS + ADHD (*N* = 17)	CDS only (*N* = 35)	ADHD only (*N* = 21)	Control (*N* = 23)	
Age (years)	22.12 (3.52)	21.03 (2.41)	21.90 (2.76)	21.61 (2.68)	0.77
Sex (male/female)	10/7	15/20	10/11	16/7	1.85
Estimated IQ	108.93 (10.88)	101.82 (12.50)	107.00 (9.98)	108.26 (9.24)	2.30
BAARS-IV					
ADHD IN	24.82 (3.75)	17.97 (2.16)	20.76 (3.90)	10.91 (3.50)	67.48*
ADHD H-I	22.59 (5.42)	14.91 (2.88)	18.86 (4.46)	10.57 (3.12)	36.60*
CDS	25.82 (2.56)	24.43 (1.56)	18.67 (3.47)	10.61 (3.87)	134.78*
ACI	29.24 (7.47)	26.34 (4.94)	20.67 (6.62)	7.65 (7.33)	50.67*
BDI-II	16.86 (11.41)	16.18 (7.54)	14.71 (10.65)	4.82 (9.63)	7.76*
BAI	15.86 (12.55)	15.29 (9.54)	12.57 (10.97)	2.64 (6.81)	8.93*
PANAS					
Positive	24.17 (6.43)	19.57 (6.07)	23.81 (6.81)	24.17 (6.43)	2.92*
Negative	24.59 (6.75)	22.17 (7.70)	22.29 (8.79)	16.52 (6.56)	4.43*

Mean (standard deviation);

*p < 0.05; Age: years, CDS, Sluggish Cognitive Tempo; ADHD, Attention-Deficit/Hyperactivity Disorder; BAARS-IV, Barkley Adult Attention-deficit/Hyperactivity Disorder Rating Scale IV; IN, inattentive; H-I, hyperactive and impulsive; CDS, cognitive disengagement syndrome; ACI, Adult Concentration Inventory; BDI-II, beck depression inventory-II; BAI, beck anxiety inventory; Estimated IQ, intelligence quotient estimated by Wechsler adult intelligence scale- IV; PANAS, positive affect and negative affect scale.

There were significant effects related to groups with regards ADHD inattention [*F*(3, 95) = 67.48, *p* < 0.01,η^2^ = 0.69], ADHD hyperactive-impulse [*F*(3, 95) = 36.60, *p* < 0.01, η^2^ 0.54], CDS [*F*(3, 95) = 134.78, *p* < 0.01, η^2^ 0.82] in BAARS-IV, and ACI [*F*(3, 95) = 50.67, *p* < 0.01, η^2^ 0.62]. The two CDS groups (CDS+ADHD, CDS only) exhibited significantly higher CDS symptoms than did the other two groups. Of other two groups, the ADHD only group presented significantly higher CDS symptoms than did Control group. In addition, the CDS+ADHD group exhibited the highest ADHD inattention and hyperactivity-impulsivity symptoms, with this finding being statistically significant. The ADHD only group also had significantly higher ADHD inattention and hyperactivity-impulsivity symptoms than did CDS only and Control group (Table 1).

In the analysis of clinical characteristics, there were significant effects related to groups with regards to BDI-II [*F*(3, 95) = 7.76, *p* < 0.01, η^2^ 0.21] and BAI [F (3, 95) = 7.76, *p* < 0.01, η^2^ 0.21]. Furthermore, the Control group presented significantly lower depression and anxiety than the other three groups. In addition, there was significant effects associated with groups for the negative affect of PANAS [*F*(3, 95) = 4.43, *p* < 0.05, η^2^ 0.13]. More precisely, the two CDS groups (CDS+ADHD, CDS only) experienced more negative affect than the other two groups (Table 1).

### 3.2 Initial orienting toward the emotional face: initial direction bias score

There was a significant interaction between CDS and emotion [*F*(2, 184) = 3.06, *p* < 0.05, η^2^ 0.03], indicating that initial direction bias differed across emotional expressions depending on CDS symptoms. *Post hoc* comparisons revealed that individuals with CDS showed a significantly lower initial direction bias toward angry faces than individuals without CDS (MD = 0.014, SE = 0.017, *p* < 0.05) (Table 2). This finding suggests that individuals with CDS symptoms were less likely to orient toward angry faces during initial attentional processing than those without CDS symptoms. There were no other significant main or interaction effects [all *ps* > 0.05]. When depression, anxiety and negative affect were included as covariates, the interaction between CDS and emotion was no longer significant, [*F*(2, 184) = 1.00, *p* > 0.05]. Neither depression, anxiety, nor negative affect significantly affected the main or interaction effects (all *p*s > 0.05).

**TABLE 2:** Comparison of initial direction bias depending on CDS and ADHD symptoms.

Measure	CDS		Non-CDS		Test statistics (*F*)
	ADHD	Non-ADHD	ADHD	Non-ADHD	
	CDS + ADHD (*N* = 17)	CDS only (*N* = 35)	ADHD only (*N* = 21)	Control (*N* = 23)	
Happy	0.55 (0.05)	0.55 (0.07)	0.54 (0.06)	0.55 (0.10)	0.12
Sad	0.55 (0.07)	0.54 (0.07)	0.56 (0.10)	0.55 (0.09)	0.23
Angry	0.52 (0.08)	0.54 (0.08)	0.60 (0.07)	0.58 (0.10)	2.97*

Mean (standard deviation);

*p < 0.05; Mean (SD) in proportion; CDS, cognitive disengagement syndrome; ADHD, Attention-Deficit/Hyperactivity Disorder.

### 3.3 Difficulty to disengage from the emotional face

#### 3.3.1 Bias score in total dwell time (proportion of time to the emotional faces)

There was a significant interaction effect between ADHD and emotion [*F*(2, 184) = 6.95, *p* < 0.05, η^2^ 0.08], indicating that participants showed difference in the dwell time to each emotional face depending on ADHD symptoms. *Post hoc* comparisons revealed that participants with ADHD exhibited significantly greater dwell time bias toward angry faces than participants without ADHD (MD = 4.158, SE = 1.588, *p* < 0.05). These findings indicate that individuals with ADHD exhibit an anger-specific attentional bias (Table 3). Otherwise, there were no other significant main and interaction effects (*all ps*). The results remained unchanged after controlling for depression, anxiety, and negative affect.

**TABLE 3:** Comparison of difficulty to disengage from the emotional face variables depending on CDS and ADHD symptoms.

Measure	CDS		Non-CDS		Test statistics (*F*)
	ADHD	Non-ADHD	ADHD	Non-ADHD	
	CDS + ADHD (*N* = 17)	CDS only (*N* = 35)	ADHD only (*N* = 21)	Control (*N* = 23)	
Bias score (proportion)					
Happy	0.54 (0.05)	0.53 (0.06)	0.52 (0.06)	0.53 (0.06)	0.55
Sad	0.51 (0.04)	0.55 (0.08)	0.51 (0.10)	0.56 (0.10)	1.89
Angry	0.57 (0.05)	0.53 (0.07)	0.56 (0.04)	0.52 (0.09)	2.29
Dwell time (seconds)					
Happy	1.23 (0.17)	1.07 (0.34)	0.98 (0.33)	0.95 (0.37)	2.77*
H-neutral	1.05 (0.20)	0.91 (0.25)	0.87 (0.27)	0.82 (0.32)	2.69
Sad	1.17 (0.18)	1.07 (0.26)	0.94 (0.30)	0.94 (0.28)	3.62*
S-neutral	1.05 (0.16)	0.94 (0.26)	0.89 (0.31)	0.85 (0.31)	1.89
Angry	1.30 (0.22)	1.07 (0.35)	1.04 (0.34)	0.94 (0.41)	3.76*
A-neutral	1.03 (0.13)	0.90 (0.27)	0.85 (0.27)	0.79 (0.36)	2.53

Mean (standard deviation);

*p < 0.05; Mean (SD) in proportion and seconds (s); CDS, cognitive disengagement syndrome; ADHD, Attention-Deficit/Hyperactivity Disorder; H-neutral, neutral faces in the pair of happy faces; S-neutral, neutral faces in the pair of sad faces; A-neutral, neutral faces in the pair of angry faces.

#### 3.3.2 Total dwell time to the emotional face

**FIGURE 2:**
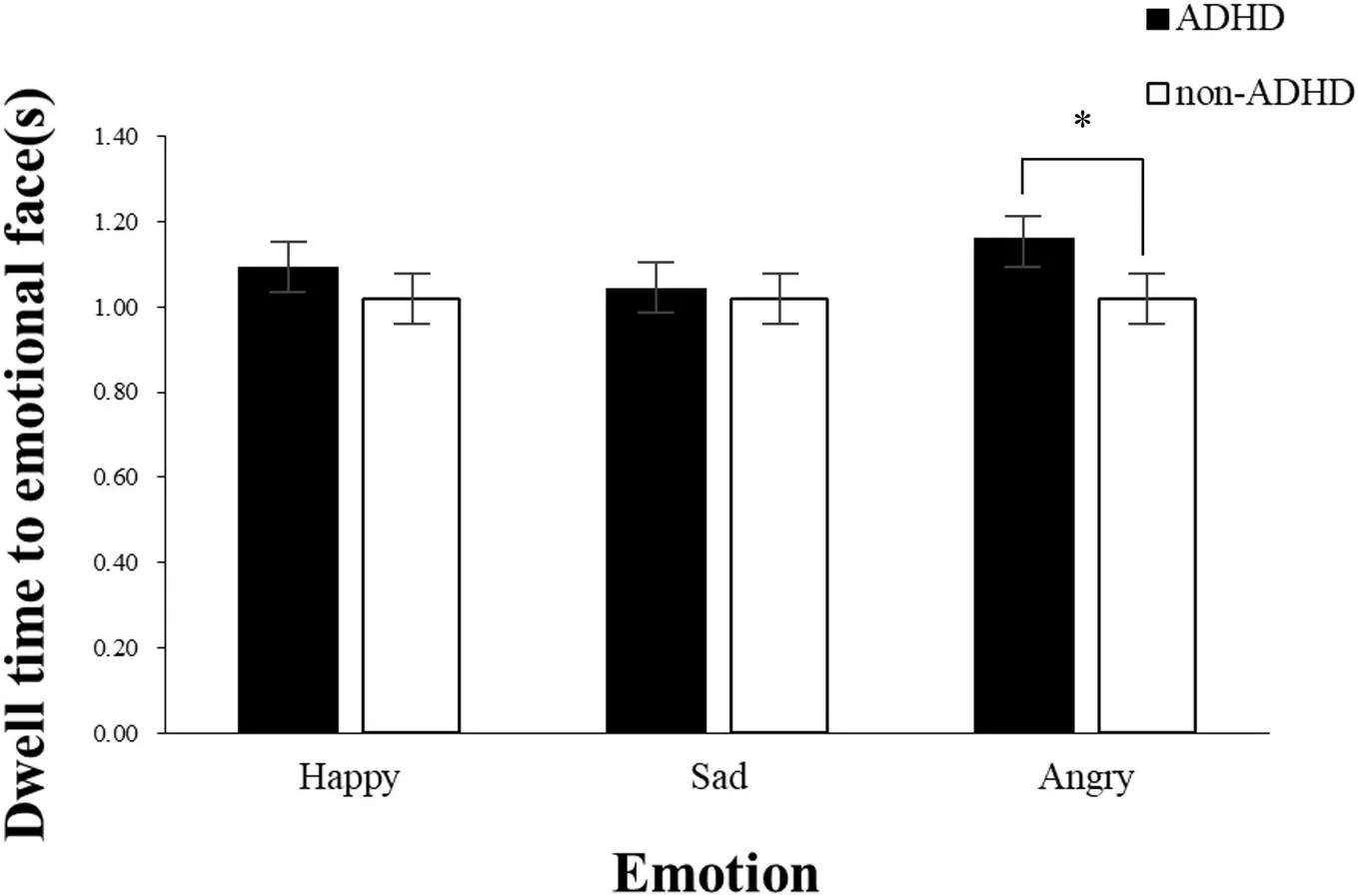
Mean dwell time to emotional face depending on ADHD symptoms (*p < .05).

**FIGURE 3:**
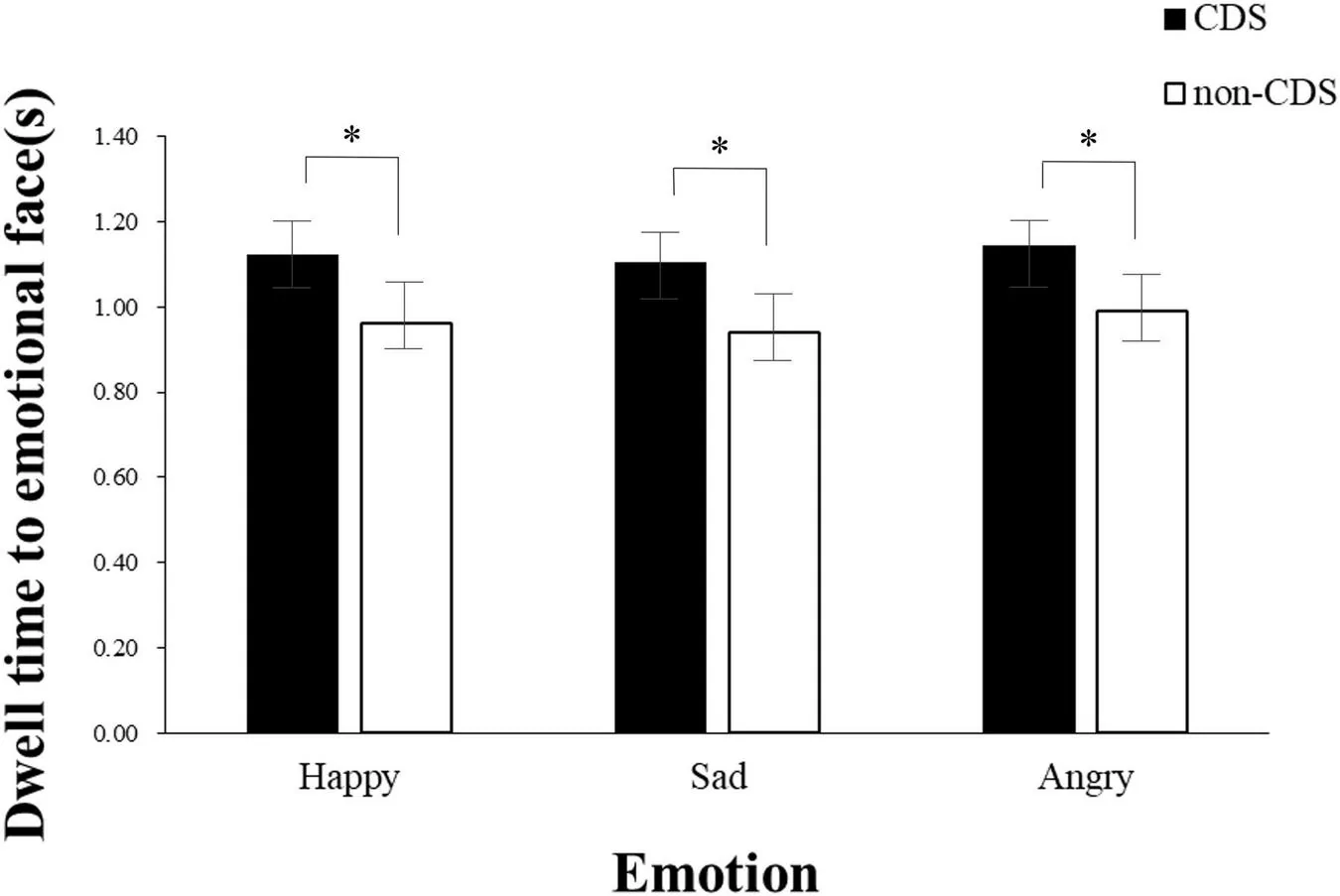
Mean dwell time to emotional face depending on CDS symptoms (*p < .05).

There was a significant interaction between ADHD and emotion [*F*(2, 184) = 7.39, *p* < 0.01, η^2^ 0.07]. Individuals with ADHD showed significantly more gaze to angry faces than those without ADHD (MD = 0.173, SE = 0.073, *p* < 0.05), thereby indicating that individuals with ADHD had a more pronounced attentional bias toward angry faces (Figure 2 and Table 3). In addition, there was a significant main effect with regards CDS [*F*(1, 92) = 8.52, *p* < 0.01, η^2^ 0.08], which was that individuals with CDS showed significantly more gaze to all emotional faces than those without CDS (MD = 0.185, SE = 0.064, *p* < 0.01). This indicated that they had increased attentional bias toward all emotional faces regardless of emotion, which in turn means they need significantly more time to process emotional faces (Figure 3 and Table 3). Also, a significant main effect of emotion was revealed [*F*(2, 184) = 6.00, *p* < 0.01, η^2^ 0.06], which showed that all participants showed significantly more gaze to angry faces than to sad faces (MD = 0.056, SE = 0.018, *p* < 0.01). The results of ANCOVA with depression, anxiety, and negative affect as covariates were the same. The pattern of results did not change.

### 3.4 The relationships between CDS symptoms and attentional indices

To investigate whether attentional characteristics was associated with CDS symptoms, a series of bivariate correlational analyses were conducted. Analyses revealed CDS symptoms of BAARS-IV were positively correlated with dwell time to sad face (*r* = 0.24, *p* < 0.05) and with dwell time to angry face (*r* = 0.23, *p* < 0.05). CDS symptoms also were positively correlated with total dwell time to all faces (*r* = 0.22, *p* < 0.05). Other correlations were also significant. ADHD-IN symptoms of BAARS-IV were positively correlated with dwell time to angry face (*r* = 0.22, *p* < 0.05). Additionally, ADHD-H/I symptoms of BAARS-IV were positively correlated with dwell time to happy face (*r* = 0.20, *p* < 0.05) and angry face (*r* = 0.26, *p* < 0.05). Table 4 showed more details in the zero-order correlation coefficients. Additional analyses using the ACI total score yielded a similar pattern of correlations. however, none of the associations reached statistical significance (Supplementary Table 1).

**TABLE 4:** The bivariate correlation between CDS symptoms and attentional indices.

Attentional indices	BAARS-IV CDS	BAARS-IV ADHD-IN	BAARS-IV ADHD H/I
Direction initial orienting - happy	0.05	−0.05	0.04
Direction initial orienting - sad	−0.07	−0.06	−0.04
Direction initial orienting - angry	−0.13	−0.11	−0.01
Dwell time to happy face	0.20	0.17	0.20*
Dwell time to sad face	0.24*	0.12	0.16
Dwell time to angry face	0.23*	0.22*	0.26*
Total dwell time to all faces	0.22*	0.19	0.19

*P < 0.05. CDS, cognitive disengagement syndrome; ADHD, Attention-Deficit/Hyperactivity Disorder; IN, inattentive; H-I, hyperactive and impulsive; BAARS-IV, Barkley Adult Attention-deficit/Hyperactivity Disorder Rating Scale IV.

## 4 Discussion

Cognitive disengagement syndrome (CDS) has been proposed within the context of ADHD research and is increasingly conceptualized as a distinct construct despite its symptomatic overlap with Attention Deficit/Hyperactivity Disorder (ADHD). However, limited evidence has been accumulated regarding whether these two conditions reflect distinct cognitive and emotional processing mechanisms. In this context, attentional processing of emotional stimuli may serve as a key indicator for examining their discriminant validity. The present study compared CDS and ADHD by distinguishing attentional bias toward emotional faces into early orienting and later attentional maintenance stages, in order to determine whether the attentional profiles of the two groups are qualitatively distinct. For the present analyses, individuals with CDS refer to participants in the CDS only and CDS+ADHD groups, whereas individuals with ADHD refer to participants in the ADHD only and CDS+ADHD groups. The findings revealed differential patterns of emotional information processing between the groups, suggesting that CDS and ADHD may be characterized by distinct underlying mechanisms.

The primary findings were as follows. First, at the early stage of attentional processing, individuals with CDS showed a reduced tendency to initially orient toward angry faces, whereas individuals with ADHD did not demonstrate this pattern. Given that CDS has been associated with hypoactivity (Miller and Prevatt, 2020), this finding may indicate inefficiency in the early process required to rapidly detect environmentally threat-related cues, such as threat-related stimuli. From an evolutionary perspective, the rapid allocation of attention to threat-related cues is considered a fundamental mechanism for facilitating appropriate perceptual and behavioral responses to potential danger (Davis and Whalen, 2001).

By contrast, individuals with ADHD exhibited early attentional responses comparable to those of typically developing adults (Fox et al., 2000). This finding was consistent with prior research indicating that ADHD does not necessarily involve deficits in the initial capture of attention by negative emotional stimuli (Ahmadi et al., 2011). Furthermore, considering that ADHD is characterized more prominently by impairments in effortful inhibitory control rather than in automatic processing (Hawk et al., 2003), the ability to detect emotional stimuli at the early stage may be relatively preserved in individuals with ADHD.

Second, a more pronounced difference emerged at the later stage of attentional processing. It was found that individuals with CDS demonstrated attentional bias across all emotional faces, whereas individuals with ADHD exhibited attentional bias selectively for angry faces. These findings suggest that CDS may not involve an emotion-specific bias, but rather a broader difficulty disengaging attention from emotionally salient stimuli. This pattern may reflect reduced flexibility in shifting attention away from emotional cues. Notably, this pattern also appears to differ from attentional biases reported in internalizing disorders. For example, depression has been associated with difficulty disengaging attention from sad faces (Sears et al., 2011), whereas anxiety disorders have been linked to avoidance patterns in response to threat-related cues (Cisler and Koster, 2010). In contrast, individuals with CDS exhibited difficulty disengaging from emotional stimuli regardless of valence. This suggests that CDS may be characterized by a distinct attentional processing profile that differs not only from ADHD but also from internalizing disorders.

Meanwhile, individuals with ADHD exhibited attentional bias selectively toward angry faces at the later stage of processing. This finding is consistent with prior research suggesting that individuals with ADHD experience difficulty effectively shifting attention in threatening contexts and show regulatory difficulties in processing anger-related information (Manoli et al., 2021). The executive function deficits and emotion regulation difficulties commonly reported in ADHD (Barkley, 2014; Harty et al., 2009) may be associated with this anger-specific attentional pattern. In addition, the positive association between total dwell time and CDS symptom severity suggests that CDS is closely related to a generalized tendency to maintain attention on emotional stimuli. This finding further supports the possibility that CDS is characterized by an attentional processing profile that is distinct from that of ADHD.

This study has several limitations. First, because CDS is not currently recognized as a formal diagnostic disorder, there may be limitations in directly comparing individuals with CDS to those with ADHD. Although our inclusion criteria required medication washout on the day prior to participation, it remains unclear whether this duration was sufficient to fully eliminate medication effects. Future studies directly comparing CDS and ADHD should adopt more stringent inclusion criteria.

Second, although the main findings remained significant after controlling for depression and anxiety, it cannot be concluded that the attentional characteristics observed in CDS cause internalizing symptoms such as depression and anxiety. Emotion and attention operate interactively, and it is possible that depression and anxiety precede, or contribute to slowed cognitive processing. However, a 2-year longitudinal study (del Mar Bernad et al., 2016) reported that elevated CDS symptoms uniquely predicted higher levels of depression and anxiety. Future research should aim to clarify the temporal ordering among anxiety, depression, and slowed attentional processing in CDS.

Third, the free-viewing task used in this study has the advantage of capturing naturalistic gaze patterns. However, because participants were not explicitly instructed to shift attention rapidly from specific stimuli, the task may not fully reflect early attentional orienting or attentional shifting abilities. Although the present findings suggest patterns of attentional allocation toward emotional stimuli, the causal relationship between these attentional patterns and real-world social difficulties was not directly tested. In a bid to better understand regulatory responses in social or conflict situations, future studies should incorporate behavioral paradigms such as go/no-go tasks. Furthermore, future studies should include time-course analyses, attentional shifting paradigms, and behavioral measures of social functioning to determine whether prolonged gaze reflects impaired attentional disengagement and to clarify its functional significance.

Finally, the correlation analyses were intended to complement the primary group comparisons and should therefore be regarded as secondary analyses. As no correction for multiple comparisons was applied, the possibility of Type I error should be considered when interpreting these findings. Accordingly, these results should be interpreted cautiously until replicated in future studies.

Despite the limitations noted above, the present study offers several important implications. Overall, the findings provide evidence for distinct patterns of attentional processing of emotional information in individuals with CDS. Specifically, individuals with CDS showed reduced initial orienting toward angry faces and a generalized attentional bias toward all emotional faces at the later stage of processing. Taken together, the present findings suggest that emotion regulation difficulties in CDS may emerge from disruptions at multiple stages of attentional processing. Specifically, reduced initial orienting toward angry faces may reflect diminished efficiency in rapidly detecting emotionally relevant environmental cues, whereas prolonged attentional maintenance across emotional expressions may reflect reduced flexibility in disengaging attention from emotional information (Cisler and Koster, 2010). From the perspective of the process model of emotion regulation, adaptive emotion regulation depends on the flexible allocation and disengagement of attention from emotional information (Gross and Thompson, 2014). Therefore, both reduced initial orienting and prolonged attentional maintenance observed in individuals with CDS may reflect reduced attentional flexibility, potentially contributing to the emotion regulation difficulties previously associated with CDS.

The observed differences between CDS and ADHD suggest that the two conditions may involve qualitatively distinct attentional difficulties. Although CDS is related to ADHD, the present findings support the view that CDS may represent a distinguishable clinical profile. Furthermore, given that CDS has been more strongly associated with internalizing symptoms, whereas ADHD has been linked to externalizing symptoms, these differing attentional patterns may contribute to their divergent emotional and behavioral outcomes.

In particular, the prolonged and generalized attentional engagement with emotional stimuli observed in CDS may be associated with difficulties in flexibly adapting to changing social demands (Srivastava and Srinivasan, 2010). Such delayed processing of emotional information could interfere with timely and effective coping in social contexts. Accordingly, these findings raise the possibility that intervention strategies targeting CDS may differ from those typically applied to ADHD, particularly in addressing internalizing symptoms and social difficulties in clinical settings. Therefore, future studies should examine whether distinct treatment approaches are warranted for CDS and ADHD.

## Data Availability

The raw data supporting the conclusions of this article will be made available by the authors, without undue reservation.
