# Supplementary material for: Attentional bias toward emotional faces in cognitive disengagement syndrome
Source: Front Psychol. 2026 Sep 7;17:1855003. doi: 10.3389/fpsyg.2026.1855003 (PMC13590327; doi:10.3389/fpsyg.2026.1855003)
Supplement: Supplementary file 1 [file Table_1.DOCX]

Supplementary Material

# 1 Supplementary Tables

Supplementary Table 1. The bivariate correlation between CDS symptoms and attentional indices

| Attentional indices | ACI | | |
| --- | --- | --- | --- |
| Direction initial orienting - happy | .02 | | |
| Direction initial orienting - sad | -.11 | | |
| Direction initial orienting - angry | -.14 | | |
|  | . |  |  |
| Dwell time to happy face | .17 | | |
| Dwell time to sad face | .17 | | |
| Dwell time to angry face | .16 | | |
|  |  |  |  |
| Total dwell time to all faces | .20 | | |

Note. **p* < .05; ACI: Adult Concentration Inventory
